# Mississippi Valley Medical Association, Hot Springs Meeting

**Published:** 1894-12

**Authors:** 


### Society Notes.

### Mississippi Valley Medical Association—Hot Springs Meeting.

[ Dr. S. E. Hudson, of the TEXAS MEDICAL JOURNAL, attended this meeting and speaks enthusiastically of it as one well attended by representative men of the profession from most or the Valley States, and very interesting. From notes furnished by him we compile the following summary of proceedings, being the first journal to give so full a report.—ED.]

### FIRST DAY—MORNING SESSION.

The convention was called to order by Dr. T. E. Holland, Chairman of the Committee on Arrangements at 11 a. m., November 20, 1894.

Invocation by Rev. Joseph A. Dickson, of Hot Springs, pastor of the First Presbyterian church.

Dr. Xenophon C. Scott, President, was introduced, who in a neat address presented Governor Wm. M. Fishback, of Arkansas.

The address of the governor was brief and concise. After alluding in a happy way to the honor conferred in addressing so august an assemblage, he dwelt upon the educational progress of Arkansas, and congratulated the medical profession that in the last two hundred years the results of their discoveries had succeeded in doubling the average length of human life. In a few happy sentences he welcomed the visitors to the State.

He was succeeded by Hon. Will H. Martin, who entertained the assemblage for ten minutes in an address of welcome in behalf of the city of Hot Springs, delivering one of the prettiest speeches of his life.

The Chairman of the Committee on Arrangements then annnounced that this evening would be "social night," when the citizens of Hot Springs would meet the visitors in social intercourse.

In the routine, the reports of the Secretary, Dr. Frederick C. Woodburn, of Indianapolis, was heard. After announcement of the Committee on Credentials, the annual address of the President was read. The address was masterly and comprehensive. In the address the President touched upon various matters pertaining to the organization, pointing towards more thoroughness in conducting the business of the Association. He alluded to the fact that this is the first time in the history of the Association that the railroads have made a one fare rate, and in behalf of the convention, thanked Col. H. C. Townsend and Mr. Chas. E. Ware for the interest they took in bringing this to pass. He outlined the moral duties of the physician, and decried the practice of consultation with "quacks" whom he designated as "legalized murderers." He urged that measures be adopted by the fraternity to the end that inventors of surgical instruments be permitted to hold patents on their inventions, and be permitted to obtain the revenue to which the inventor's skill entitles him.

After motion for the appointment of a committee on president's address, the convention adjourned until 2 p. m., sharp.

AFTERNOON SESSION.

In the afternoon the first paper read was by Dr. W. S. Keer, of Mansfield, O.—"Some observations on the rights and duties of medical witnesses." In this paper the reader outlined the difficulties under which expert testimony is given from the lawyer's standpoint. This was the only paper on medical jurisprudence that was read at this convention. The paper was discussed by Dr. Harold N. Moyer, of Chicago, who elucidated some of the shortcomings of the legal fraternity, deducing his observations from extensive personal experience. The discussion was continued by Dr. Hughes, of St. Louis, and Kirby, of Harrison, Ark. The point at issue was as to the matter of compensation for such testimony.

The second paper was "Bone and joint tuberculosis the future field of litigation against railways," by Emory Lanphear, of St. Louis. The burden of the argument was that minor sprains are frequeutly primary cause of tuberculosis; that tuberculosis is not inherited, but always acquired.

Several papers down on the program were not read on account of the absence of the gentlemen who were to read them.

The next paper was by Robert H. Babcock, of Chicago, on "Enlargement of the heart without valvular disease, with special reference to treatment." This paper was highly scientific, and exceedingly interesting, heightened by the fact that the speaker is entirely blind.

"Oxygen as a heart tonic and some of the benefits which may be derived from its use as such," was the third paper, read by W. T. Baird, M. D., of Dallas, Texas.

Upon motion of Dr. Stuckey, of Kentucky, Dr. W. H. Daily, of Pittsburg, was permitted to read a voluntary paper, "Malaria, a water-born disease," the object of the argument being to establish by experience and proofs by observation that malarial diseases are contracted only by drinking malaria infected water. Dr. Hughes in the discussion that ensued insisted that it is also a vapor-born disease and cited his own experience in substantiation that vapor is the carrier of malaria. In his response Dr. Daily said that without being a prophet, or the son of a prophet, neither the seventh son of a seventh son, he felt confident in making the assertion that within five years the idea that now prevails relating to the matter of conveyance of malarial germs by the atmosphere would be consigned with other exploded theories to the realms of d—n nonsense.

The concluding paper of the day's program was read by Dr. A. Ravogli, the subject being "The influence of early treatment on late manifestations of syphilis." It was a comprehensive paper, scientific and advocated the earliest constitutional treatment after the appearance of symptom, as the initial lesion is no criterion as to the gravity of the lesion.

SECOND DAY—MORNING SESSION.

Little time was occupied this morning in preliminory work before the convention got down to work. The report of the Treasurer, Dr. George J. Cook, of Indianapolis, was read by the Secretary.

Dr. J. M. Ball, of St. Louis, was given permission to make an announcement, and he extended an invitation to the members of the convention to attend a meeting of the Missouri, Illinois and Iowa Tri-State Medical Convention, which meets in St. Louis the first Tuesday, Wednesday and Thursday in April, 1895.

The first paper read in the morning programme was "Intestinal Indigestion," by Dr. A. P. Buchannon, of Fort Wayne, Ind.

The chairman announced the names of the Committee on President's Address—Drs. W. W. Patten, Buffalo, chairman; I. N. Love, St. Louis; Thos. Hunt Stuckey, Louisville, Ky.; A. S. Garnett, Hot Springs, and C. B. Parker, Cleveland.

Dr. A. M. Owen, chairman of the Committee on Credentials, read his report, after which the last paper read was discussed at considerable length.

The next paper was by Dr. J. C. Woodbridge, of Youngstown, O., "Typhoid fever can be aborted; another year's work with no death and no failure in evidence." The paper, a lengthy one, was followed by another, "Ox-gall treatment in typhoid," and inaugurated the most interesting part of the morning's discussion. In his paper Dr. Woodbridge assumed the position that any case of typhoid fever could be aborted in from six to twelve days. These papers elicited the greatest interest and discussion since the meeting convened. The consensus of opinion was antagonistic to the views expressed by Dr. Woodbridge.

Dr. Love, the first speaker, censured in severe terms the dogmatic position assumed by the author in stating that "any physician who lost a patient from typhoid fever should be sued for malpractice."

After elucidating the changes taking place in the intestines during the fever, Dr. Hamilton pointed out the inaccuracies in the tabulated reports submitted.

Dr. Porter, of Texas, gave as his experience of forty years that the conditions contributing to produce the typhoid varied as to the climate, habits and surroundings.

Dr. G. Frank Lydston, of Chicago, pointed out the importance of correct diagnosis—that to do this, time was necessary, it being impossible to make this diagnosis in one or two visits.

Dr. Loving, of Ohio, made a very interesting and witty speech, concluding that the principal treatment was to "cool the patient when hot, to warm him when cool, to stimulate when weak, to tranquilize when wakeful."

Dr. Woodbridge, in conclusion, took exceptions and made strong objections to the outrageous manner in which he said he had been treated. He declared that time alone would confirm the correctness of his views.

Interesting discussion of the paper on "The importance of urinalysis in diagnosis," by Dr. A. B. Walker, of Canton, O.; and "My experience with gold as a therapeutic agent," by Dr. A. M. Owen, of Evansville, Ind.

### AFTERNOON SESSION.

At the beginning of the afternoon session the Nominating Committee were announced: Drs. Love, St. Louis; Lydston, Chicago; Cook, Indianapolis; Potter, Texas; Collings, Hot Springs; Walker, Detroit; Coffin, Kansas City; Barklay, Pittsburg; Holland, Hot Springs.

The first paper of the afternoon was by Dr. Sterling Loving, of Columbus, O., "Physicians' Prescriptions." The paper dwelt upon the carelessness of physicians in their chirography in writing prescriptions.

"Toxics," by Dr. Wm. F. Barklay, of Pittsburg, was a scholarly and scientific paper.

"Quinine in Chorea," was the succeeding paper, read by Dr. Frank R. Fry, St. Louis. This paper was discussed at considerable length by Drs. Hughes, of St. Louis; Harold Moyer, of Chicago; Ricketts, of Cincinnati; Ashton, of Texas, and others.

"Reflex Irritation as a Cause of Nervous Diseases," was the subject of an interesting paper by Dr. Edwin Walker, of Evansville, Ind., which was also discussed at considerable length.

An exceedingly interesting and entertaining paper was that of Dr. Harold N. Moyer, of Chicago, his subject being "Accidents and Injuries from Electric Currents of High Potential." It was scientific and betrayed great research and observation.

### THIRD DAY—MORNING SESSION.

Less than one hundred members of the convention were in attendance when the gavel of the President fell this morning.

After calling the body to order, the secretary read the following telegram:

DETROIT, MICH., November 22, 1894.

*President Scott, Mississippi Valley Medical Association, Hot Springs, Arkansas:*

I am unavoidably detained at the last moment; am greatly disappointed. My warmest wishes and best regards for your meeting. Make each member swear to meet me in Baltimore next May, alive or dead.

DONALD MCLEAN,
President American Medical Association.

The first paper of this morning's session was that of Dr. Frank P. Norbury, of Jacksonville, Illinois, subject: "The Mental Symptoms of Cerebral Syphilis, a Clinical Study." The paper was an interesting one, but elicited no discussion.

The next paper—"The Surgical Treatment of Injuries of the Head"—by Dr. Chas. B. Parker, of Cleveland, Ohio, suggesting the necessity of exploratory incisions, to investigate certain unfavorable symptoms, and citing interesting experiences. The paper was discussed by Drs. Link, of Indiana, Ricketts, of Cincinnati, Minney, of Topeka, and Walter, of Detroit.

Dr. Geo. N. Lowe was excused from reading his paper—"Spot Specialism"--except by title, on the grounds of just having had his teeth drawn, and consequent difficulty of enunciation.

Dr. B. Merrill Ricketts, of Cincinnati, made a "Report of cases (a), castration for Hypertrophied Prostrate; (b) removal of head of femur for dislocation into lesser sciatic notch; (c) trephine for pressure as a result of fluid in acute cerebral meningitis." Discussion by Drs. Moyer, Walker, of Detroit, Walker of Evansville, and others.

At the close of the discussion, Chairman of Arrangements, Dr. Holland, announced that the hot springs on the Hot Springs mountains would be open to the inspection of the members of the convention during this afternoon. Also, that Dr. Garnett and lady would hold a reception at their residence from 3 to 7 o'clock this afternoon, and that the ladies of the city were preparing an entertainment for the visiting ladies at the Arlington to-night.

The banquet at the Park was announced to be for gentlemen only, the banquet to begin at 8:30 o'clock.

The next paper was read by Dr. William E. Wirt, of Cleveland, Ohio,—"Tumor Albus of the Knee Joint."

Before proceeding to discussion of the paper that of Dr. Meisenback, of St. Lonis—"Resection of the Knee for Separation of the Lower Epiphysis of the Femur, a case of two years' standing in a patient thirteen years old" —was called for and read as being of the same class as the preceding paper. This was illustrated with charts, drawings and casts. The papers brought out a prolonged and interesting discussion, participated in by Drs. Ricketts, Lanphear and Link.

"Colles Fracture" was the subject of an interesting paper by Dr. J. E. Link, of Terre Haute, Indiana. Dr. Link, during the reading, exemplified his process of operating in the reduction of the fracture and bandaging, by performing the actual work on a subject before the convention. After an animated discussion the convention adjourned until 2:30 p. m.

### AFTERNOON SESSION.

Immediately after the convention was called to order in the afternoon, Dr. J. H. Kellogg, of the Battle Creek Sanitarium, made a brief talk, illustrated by charts, of deformities of women.

At the conclusion of his talk, Dr. C. R. Holmes, of Cincinnati, read a paper—"Diseases of the Accessory Nasal Cavities; their Influence upon the Organs of Sight; Modern Surgical Treatment, with report of cases." This was the most interesting paper of the afternoon, and was illustrated by a series of charts, and over a score of parts of skulls showing the frontal sinus exemplifying the results of diseases in the portions of the head. Through the courtesy of Dr. Loeb, granting the essayist his time, Dr. Holmes occupied forty minutes, and was heard with the utmost interest, as a number of new ideas were elucidated which met the approval of the convention. A protracted discussion followed the close of Dr. Holmes' elucidation, participated in by a large number of the physicians in attendance.

"Hydrocele" was the scholarly paper that followed, by Dr. W. C. Weber, of Cleveland, Ohio.

"Case of Traumatic Cataract in Children, Treated by Extraction," was an instructive paper by Dr. James M. Ball, of St. Louis.

"Some Observations on Tonsils" was the subject of a most instructive paper by Dr. L. C. Cline, of Indianapolis. This was one of the most important papers presented, inasmuch as it elucidated some new ideas as to the cause and for the cure of the troubles specified. The experience of the essayist was that in cases where tuberculosis was suspected from the nature of offensive cheese-like globules coughed out, where alarming symptoms existed, and quinzy accompanied, the sole cause of the inflammation of the tonsil resulted simply from these deposits which, removed, and the sacs destroyed, the trouble ceases. The discussion following brought out confirmatory expressions.

"Squint, with Special Reference to an Operation," was a highly scientific paper by Dr. Chas. Beard, of Chicago, who illustrated the operarion by original blackboard drawings.

The occasion closed, of course, with a grand banquet, at which the irrepressible Love was toastmaster. The *Sentinel* says, "he interlarded" the toasts. We infer, therefore, they must have been very *dry*. The only objection we have to the banquet is, the menu was printed in dog French, and a poor article of it, at that.

